# Print evaluation of inks with stealth nanobeacons

**DOI:** 10.1039/d4ra08210a

**Published:** 2025-02-10

**Authors:** Akinobu Yamaguchi, Toshiya Yasunaga, Kyoko Namura, Motofumi Suzuki, Takao Fukuoka

**Affiliations:** a Department of Electrical, Electronic and Communications Engineering, Faculty of Science and Engineering, Toyo University 2100 Kujirai Kawagoe Saitama 350-8585 Japan yamaguchi054@toyo.jp; b Laboratory of Pharmaceutical Engineering, School of Pharmacy, Aichi Gakuin University 1-100 Kusumoto-cho, Chikusa-ku Nagoya 464-8650 Japan; c Department of Micro Engineering, Kyoto University Kyoto Daigaku-Katsura, Nishikyo-ku Kyoto 615-8540 Japan; d Archilys Corporation, Advanced Science, Technology and Management Research Institute of Kyoto 8E09, 8F, 134 Chūdōji Minamimachi, Shimogyo-ku Kyoto 600-8813 Japan

## Abstract

Plasmonic structures using noble metal nano-assemblies are created and printed or stamped with a seal for use as information tags that carry both authenticity and information. We created an ink that contains stealth nanobeacons and evaluated its printing characteristics. Stealth nanobeacons are composed of noble metal nano-assemblies, which are fabricated *via* a self-assembly process and have indefinite shapes. This plasmonic structure was made into simple ink by mixing it with pure water or existing inkjet printer inks. We discharged this adjusted ink on an inkjet printer to evaluate its surface-enhanced Raman scattering activity and other properties, and confirmed that the ink containing stealth nanobeacons can be printed successfully. The printable ink is expected to be developed into a “Nanotag” information tag and an authenticity tag.

## Abstract 2

Plasmonic structures using noble metal nano-assemblies are created and printed or stamped with a seal for use as information tags that carry both authenticity and information.

## Graphical abstract

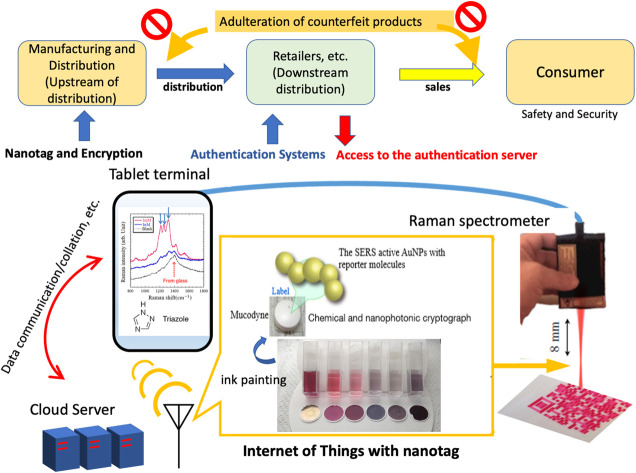

## Introduction

With the rise of e-commerce, counterfeit and imitation products are becoming a growing problem worldwide.^1–7^ For example, counterfeit medicines pose health risks and may endanger life. There have been many reports of counterfeit medicines such as malaria drugs and Viagra because of the high levels of trading in these medicines.^5–11^ To fight counterfeiting, various anticounterfeiting technologies have been researched and developed around the world.^12–50^ The reason for the entry of these counterfeit products into the market is that there are opportunities for counterfeiters to enter distribution and product management systems that are controlled using tags and labels, which are generally paper-based. The various anticounterfeit tags that are currently available frequently rely on the use of deterministic processes to create images or barcodes. Here, the deterministic process is a process that builds or operates a system in which no randomness is involved in the unfolding of future states of the system. The process operates by means of a deterministic model and always produces the same output from a given starting condition or initial state. For example, a barcode or QR code has a one-to-one correspondence of one pattern to one character or string of characters, and printing the pattern, for example, will always produce the information corresponding to that pattern. Therefore, to protect against this security concern, the generation of more complex tags is being promoted, but the complexity required is often accompanied by high costs for fabrication and analysis.

In recent years, research and development efforts to combat these counterfeiting problems have been made, but the problems described above with the forms of these tags and labels mean that many have yet to leave the research and development stage. To overcome this situation, the development and introduction of taggant technology has been conducted. In particular, “Microtaggants” which are small and difficult to detect using the naked eye at first glance have attracted much attention because these taggants offer the ability to provide tracking additives that can be incorporated into products to identify authentic products, in forms such as radio frequency identification (RFID) devices,^19^ deoxyribonucleic acid (DNA) inks,^13,20^ specialized chemicals,^12,18^ and other unique identification substances.^17,18,21–50^ These methods and techniques, however, are generally visible and can be stripped, reused, imitated, and reverse engineered. In addition, the DNA inks are laboratory-based, do not react instantaneously for detection, reading and authentication applications, and also require specific sample preparation methods and confirmation of medical safety.

Although Raman scattering has been used as a powerful chemical fingerprinting technique, it is a comparatively weak signal and is not perceived to be practical for use with taggant technology. Recently, we have developed stealth nanobeacons composed of self-assemblies of colloidal gold nanoparticles with reporter molecules.^23,24,26,27^ These stealth nanobeacons produce characteristic surface-enhanced Raman scattering (SERS) activity^51–57^ with long-term stability.^24^ In our previous study,^24^ distinguishable SERS signals were able to be detected rapidly from the reporting molecules when the nanobeacons were deposited on a very tiny area on commercial tablets. This functionality alone can serve as a nanotag, but to allow the nanobeacons to carry more complex information, it is believed that combination of the nanobeacons with pattern printing using ink is necessary. If such a stealth nanobeacon can be inked and printed, it can then carry a great deal of information and can be incorporated into authentication systems. Inkjet printing using nanoparticles has been reported by several groups^58–61^ and it has been demonstrated that it is possible to print nanoparticles such that they exhibit SERS activity.^60,61^ In this work, we prepare inks containing the nanobeacons and then inkjet print them to investigate their printing properties.

**Fig. 1:**
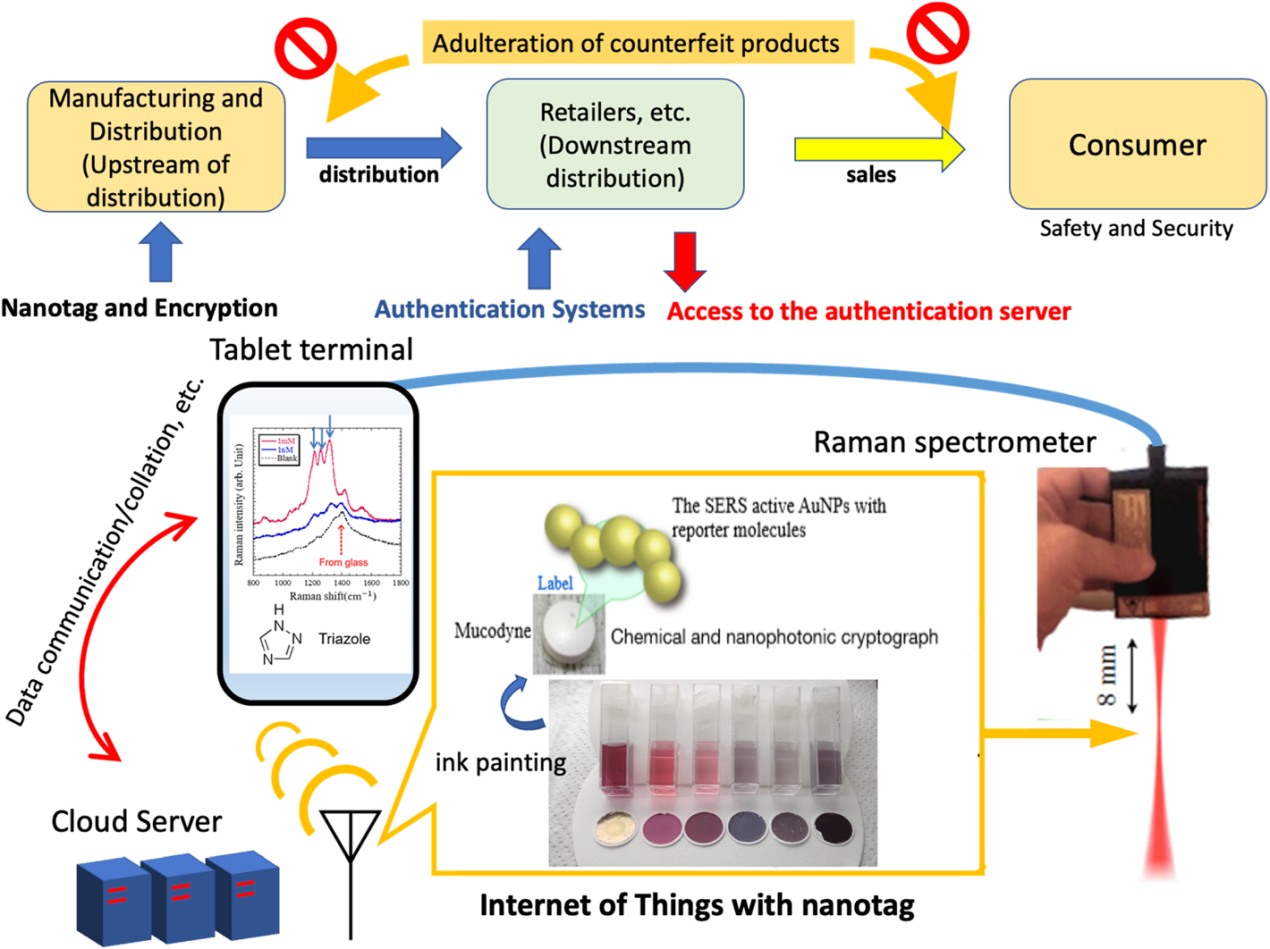
Schematic of authentication systems based on nanotags for logistics from manufacturing to distribution, retailers, sales, resales, and the consumer, providing safety and security.

In the study, we fabricate an ink by mixing normal inkjet printing ink with the stealth nanobeacons and then attempt to print it and evaluate the print characteristics that are realized. If our research and development of this technology progresses appropriately, as shown in Fig. 1, product information that is labeled at the very top of the manufacturing and distribution stream, and that combines digital data with product data in a tag in a one-to-one manner, will make it possible to prevent counterfeit products from being mixed among the genuine items through subsequent distribution channels. In addition, this nanotag authentication system also can provide a digital product passport. Fig. 1 shows a schematic diagram of how these nanotags can be used as part of the social infrastructure. To enable nanotagging to advance, this study reports on the properties of stealth nanobeacons that are inked and then printed using an inkjet printer.

## Materials and methods

Stealth nanobeacons were prepared by mixing 4,4′-bipyridine (4bpy) with gold nanoparticles that had been prepared *via* a citric acid reduction of an aqueous gold chloride solution.^23,24^ Nanobeacons are nanoparticle aggregates with indefinite shapes that are dispersed in a metastable state in solution. Because there may be cases in which the nanobeacons form clusters with each other or the nanobeacons are larger in size, they were crushed and homogenized using an ultrasonic crushing homogenizer process. The ultrasonic oscillating element homogenizer NR-50M (Microtec Co., Ltd, Chiba, Japan) was used for 10 min. Two solution types containing the adjusted stealth nanobeacons were prepared: (1) stealth nanobeacon solution : ultrapure water = 1 : 1, and (2) stealth nanobeacon homogenized solution : ultrapure water = 1 : 1. In addition, we also prepared inks by mixing the yellow and magenta inks from a commercial inkjet printer (Canon Inc.) with the homogenized stealth nanobeacon solution at a ratio of 1 : 1. Their descriptions are summarized here and the adjusted inks are denoted by A, B, C, and D, where: A represents NB : ultrapure water = 1 : 1 without homogenization; B represents NB : ultrapure water = 1 : 1 with homogenization; C represents NB : yellow = 1 : 1 with homogenization; and D represents NB : magenta = 1 : 1 with homogenization, where NB denotes the stealth nanobeacon solution. The ink compositions are summarized in Table 1. Printing was performed using an inkjet printer (Cluster Technology, Co., Ltd, Osaka, Japan; *ϕ* 15 μm nozzle), and the ways in which the intensity and the spatial distribution of the surface-enhanced Raman signal varied based on the printing conditions were investigated *via* a specially improved Raman microspectroscopy system (Nikon FN-1 microscope with a Lambda Vision Inc., Kanagawa, RAMS300S system; excitation wavelength: 785 nm; power: 200 mW; 50× objective lens; stage accuracy of less than 1 μm; exposure time to measure one point: 100 ms, which the Raman signal from the nanobeacon is sufficiently detectable while the Raman signal from the ink itself is at an undetectable time). The printing conditions for the inkjet printer were set at a *ϕ* 15 μm nozzle diameter, a 20 V discharge voltage, and a 1 kHz cycle rate, and the number of shots (*i.e.*, the number of times that a spot is printed) was adjusted. The volume of a single droplet was approximately 4.2 pL. All inkjet printing experiments and observation evaluations were conducted at room temperature in atmospheric air.

**Table 1:** Summary of the properties of the inks prepared in this study^a^

Label	Mixing ratio	Treatment
A	NB : ultrapure water = 1 : 1	Without homogenization
B	NB : ultrapure water = 1 : 1	With homogenization
C	NB : yellow = 1 : 1	With homogenization
D	NB : magenta = 1 : 1	With homogenization

a NB denotes the nanobeacon solution.

## Results and discussion

**Fig. 2:**
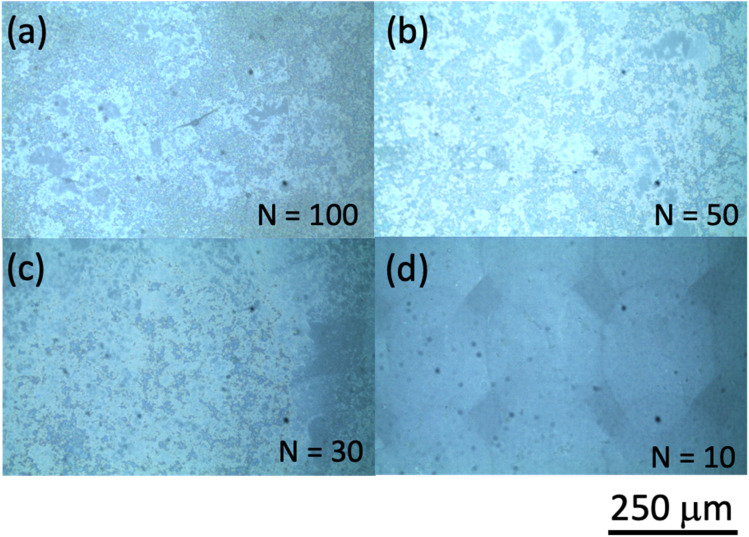
Optical microphotographs of areas printed using ink ‘A’ with number of shots *N* of (a) 100, (b) 50, (c) 30, and (d) 10. The scale bar length is 250 μm.

Our inkjet printing using the Cluster technology printer was performed on photo paper for use with an inkjet printer from Canon Inc. (basis weight: 265 g m^−2^; paper thickness: 0.27 mm; International Organization for Standardization (ISO) brightness: 92%). Some results of inkjet printing using ink ‘A’ are shown in Fig. 2, where we show optical microscope images for the number of shots *N* = (a) 100, (b) 50, (c) 30, and (d) 10 to examine the dependence of the printing characteristics on *N*. When *N* is 10, the shapes of the printed dots can be discerned. However, as the number of shots increases, the dot shape structures can no longer be distinguished. This is believed to be caused by the fact that as the number of shots increases, the droplets are dropped one after another before the previously ejected droplets can dry, with larger droplet sizes forming on the paper surface as a result. As the number of shots increases, the nanoparticle aggregates in the ink may also agglomerate and become spatially distributed, and the pattern is considered to appear as if a nonuniform structure has spread over the entire surface. Here, we should consider the following thing. How much nanobeacons are spatially dispersed per unit area is determined by the number of nanobeacons in the ink and the printing conditions? The area occupied by nanobeacons per unit area becomes larger as the number of nanobeacons dispensed increases. However, the coverage per unit area changes depending on whether it is evaluated as the percentage occupied as particle distribution or as the spread of SERS emission intensity. Here, the spatial spread of SERS emission intensity is evaluated as a printing characteristic of nanobeacon ink per unit area. As an indicator, we examined the percentage of coverage of the SERS emission distribution by the number of dispensing times.

**Fig. 3:**
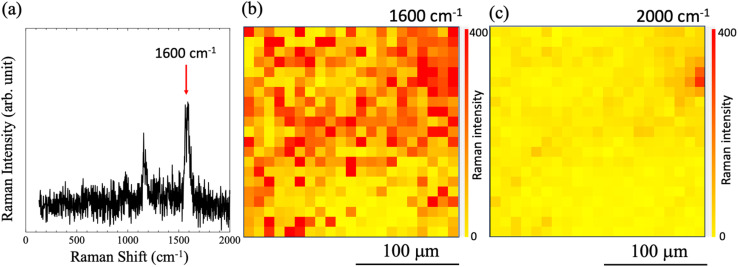
(a) Typical surface enhanced Raman scattering (SERS) spectrum of an area printed using ink ‘A’, which was generated from nanobeacons that included 4,4′-bipyridine (4bpy). The Raman shift of 1600 cm^−1^ was derived from stretching of the benzene ring of 4bpy. Spatial mappings of the Raman intensities at (b) 1600 cm^−1^ and (c) 2000 cm^−1^, where the observation area was 200 μm × 200 μm and mappings were performed using a step size of 10 μm.

Micro-Raman mapping was performed over the printed area. Some of the observations from the micro-Raman mapping procedure at *N* = 100 shots are shown in Fig. 3. The typical SERS spectrum generated from the nanobeacons, including 4bpy, is shown in Fig. 3(a). The Raman shift of 1600 cm^−1^ is derived from the stretching of the benzene ring of 4bpy.^62–65^ Spatial mappings of the Raman intensities at 1600 cm^−1^ and 2000 cm^−1^ are shown in Fig. 3(b) and (c), respectively, where the observation area in both cases was 200 μm × 200 μm and the mappings were performed using a step size of 10 μm. As a result, a highly intense Raman peak at 1600 cm^−1^ is distributed over the entire observation area, whereas no high-intensity distributions are observed in the mapping at 2000 cm^−1^. In Fig. 3(c), there is a point in the upper right of the image where the intensity of the 2000 cm^−1^ peak is higher than that in the other areas, but at the corresponding location in Fig. 3(b), the intensity of the 1600 cm^−1^ peak is also clumped significantly. Here, because the Raman peak at 2000 cm^−1^ is not a signal that originated from the nanobeacons but is in fact the baseline of the spectrum, the area in which the intensity distribution is higher suggests that a clump of large-sized aggregates consisting of nano-aggregates (*i.e.*, nanobeacons) is present at this location. This is because the presence of clumps of nanobeacon aggregates will cause the focal point to shift due to the height change or the baseline to change because of photoluminescence.^66,67^ The SERS intensification strength also strongly depends on the shape of the aggregate structure and the number of particles. As reported in more detail in the references of 56 and 57, the laser absorption wavelength varies with particle size and number of chains, and aggregate structure with resonance structures that are well matched to the resonance wavelength of the molecule show higher SERS intensity. In addition, for example, 3D porous structures may behave like photonic crystals and may exhibit photoluminescence changes and optical confinement effects. These combined effects could result in a secondary increase in photoluminescence, *etc.*, when nanobeacon aggregates are stacked, which could be superimposed on the Raman signal baseline.^66,67^

**Fig. 4:**
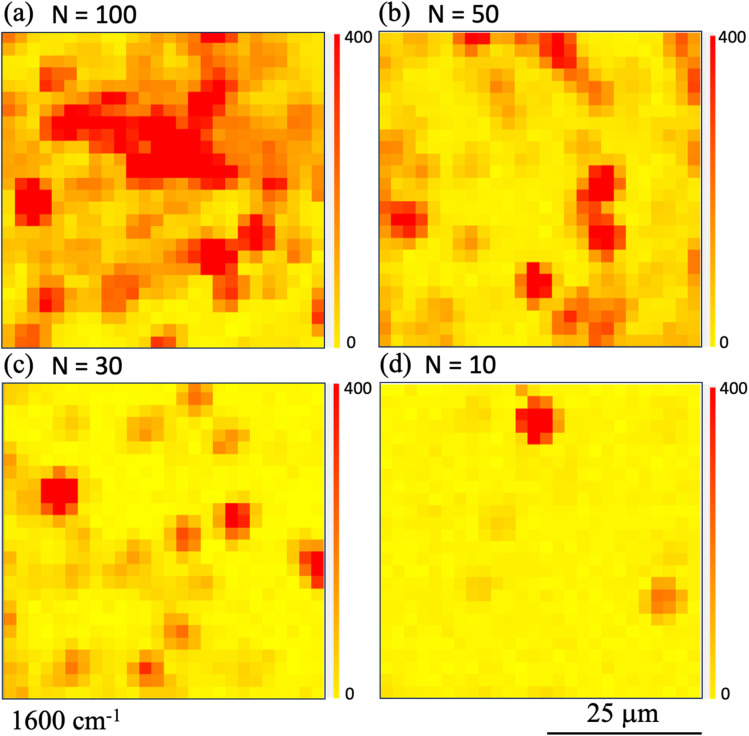
Raman intensity mapping images obtained in cases where the shot number *N* is (a) 100, (b) 50, (c) 30, and (d) 10, when printing using ink ‘A’. Here, the observation area was set at 50 μm × 50 μm with measurements being taken using a step size of 2.5 μm. Raman mapping images acquired at 1600 cm^−1^ are shown. The red (yellow) color denotes the high (low) Raman intensity.

To determine the shot number dependence of the SERS signal's spatial distribution, we compared the Raman intensity mappings of the printed areas at 1600 cm^−1^. Fig. 4(a)–(d) show the Raman intensity mapping images obtained in the cases where the shot number *N* was (a) 100, (b) 50, (c) 30, and (d) 10, respectively. Here, the observation area was set to have dimensions of 50 μm × 50 μm and was measured using a step size of 2.5 μm. As expected, the area of high Raman intensity increased as *N* increased.

**Fig. 5:**
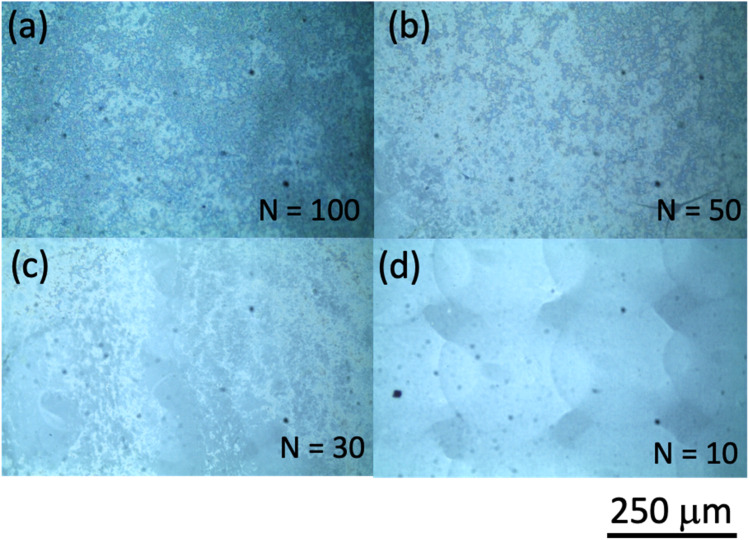
Optical microphotographs of printed areas using ink ‘B’ with number of shots *N* of (a) 100, (b) 50, (c) 30, and (d) 10. The scale bar length is 250 μm.

**Fig. 6:**
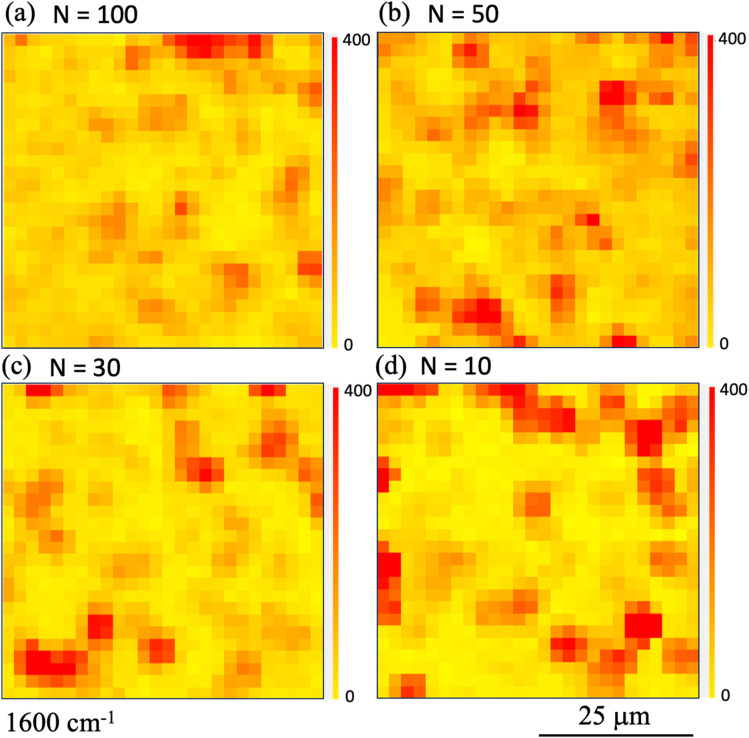
Raman intensity mapping images obtained in cases where the shot number *N* is (a) 100, (b) 50, (c) 30, and (d) 10, when printing using ink ‘B’. Here, the observation area was set at 50 μm × 50 μm with measurements being taken at a step size of 2.5 μm. Raman mapping images acquired at 1600 cm^−1^ are shown. The red (yellow) color indicates the high (low) Raman intensity.

Next, to compare the ways in which the printing properties change depending on the dispersion conditions of the nano-assemblies in the ink, we evaluate the printing performance of nanobeacon ink ‘B’, which was pretreated using an ultrasonic homogenizer. Fig. 5 shows optical micrographs acquired from the printed area under conditions where the number of shots *N* was (a) 100, (b) 50, (c) 30, and (d) 10. The results obtained do not appear to change a great deal from those shown in Fig. 2. In the same manner as before, microscopic Raman mapping images were obtained and the results are shown in Fig. 6. The observation area was the same as that in Fig. 4 at 50 μm × 50 μm, with measurements being taken at a step size of 2.5 μm. The results show that the area of high Raman intensity is spread and distributed almost uniformly for all shot numbers. When compared with the cases without homogenization shown in Fig. 4, these results are not so strongly dependent on the number of shots. As the number of shots increases, the number of nanobeacons present per unit area should also increase accordingly. The number of hotspots with SERS activity does not appear to increase based on the Raman mapping observations in Fig. 6, as described above. As we will discuss later, Raman mapping image analysis of the intensity distributions indicates that the number of hotspots increases with increasing numbers of shots. The result for the *N* = 100 case is regarded as the distribution of nanobeacons that happens to be slightly spatially biased within the observation area.

**Fig. 7:**
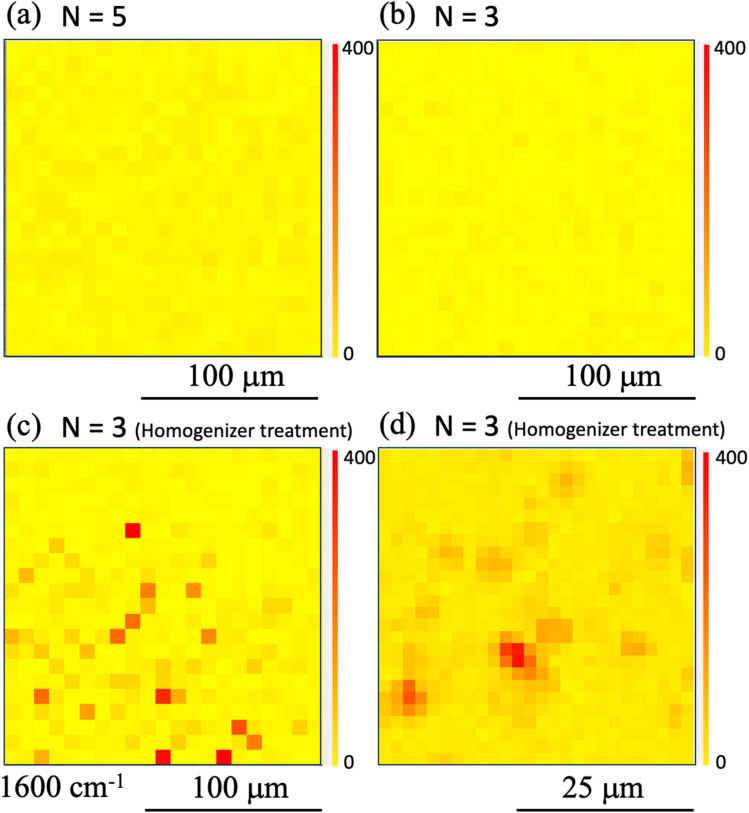
Raman intensity mapping images obtained in the case of printing using ink ‘A’ with the number of shots of (a) *N* = 5 and (b) *N* = 3. The observation area for these two mapping images is 50 μm × 50 μm with measurements being taken at a step size of 2.5 μm. Raman intensity mapping images of areas of (c) 200 μm × 200 μm with measurements being taken at a step size of 10 μm, and (d) 50 μm × 50 μm with measurements being taken at a step size of 2.5 μm, after printing using ink ‘B’ with the homogenizer treatment. Raman mapping images at 1600 cm^−1^ are also shown. The red (yellow) color denotes the high (low) Raman intensity.

**Fig. 8:**
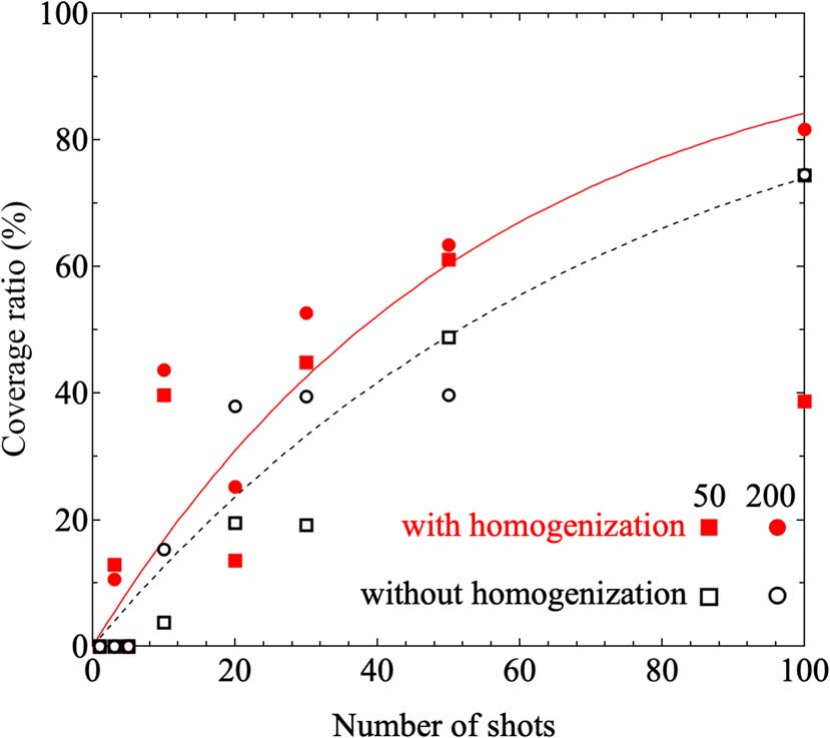
Shot number dependence of the coverage ratio in the observation area when printed using inks ‘A’ and ‘B’. Here, ‘A’ and ‘B’ are inks without and with homogenizing treatment, respectively. The red (white) squares and circles denote the coverage ratios estimated in the cases of the observation areas with dimensions of 50 μm × 50 μm and 200 μm × 200 μm, respectively, with (without) the homogenizing treatment. The black dashed line and the red solid line correspond to the fitting results for the coverage ratios printed using inks ‘A’ and ‘B’, respectively, when fitting is performed using eqn (2).

Furthermore, results obtained under printing conditions with a reduced number of shots for comparison are shown in Fig. 7. These Raman intensity images were mapped for the Raman shift of 1600 cm^−1^. Fig. 7(a) and (b) show the results for shot numbers of (a) *N* = 5 and (b) *N* = 3 in the case without homogenization. As the mapping results show, no locations with high Raman intensity emissions were observed in the mapped region. In contrast, the results of Raman mapping for the number of shots *N* = 3 after homogenization are shown in Fig. 7(c). Several locations with high Raman intensity emissions were observed and these locations appear to be distributed over the entire observation area. Fig. 7(d) shows the results of Raman mapping at higher magnification when compared with the results shown in Fig. 7(c). This figure shows that the Raman peaks that originated from the SERS signals from the nanobeacons do indeed appear to be uniformly distributed in spatial terms. The shot number dependence of the coverage ratio of these Raman intensity mapping images is summarized in Fig. 8. The relevant data analysis was performed using ImageJ software.^68^ In the ImageJ analysis, the area was calculated by binarizing the spectral intensity with respect to a threshold value based on whether the spectral intensity is exhibited or the background signal with no peak intensity is exhibited. The results in Fig. 8 show again that the homogenizer treatment tends to lead to higher coverage overall. Here, the indexes 50 and 200 represent the observed areas of 50 × 50 and 200 × 200 μm^2^, respectively. When the ink containing the nanobeacons is ejected by the inkjet printer, droplets that randomly contain nanobeacons are dropped onto the paper and the nanobeacons then adhere within the printed area.

As the number of shots is increased, this printing process occurs periodically at specific time intervals. The nanobeacons contained in each dispensed droplet are assumed to be independent and to be distributed randomly in both the droplet and the printed area. The characteristics of such a single dispensing step can be described well using a random number that follows an exponential distribution, *i.e.*, an exponential random number. The exponential distribution function is a continuous-type probability distribution, and is the distribution that describes the period of time between one event and the next, *e.g.*, the period of time between the occurrence of a disaster and the next occurrence. The probability density function is represented by the following equation:1
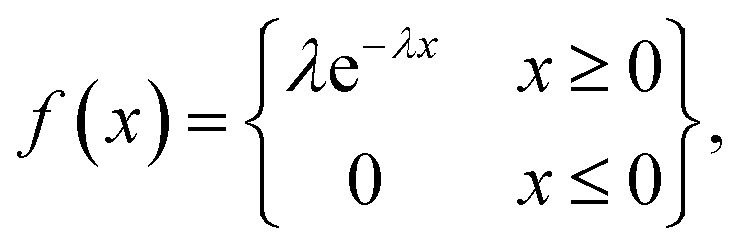
where if a phenomenon occurs *λ* times on average within a certain time period and the period *X* = *x* until the next occurrence follows an exponential distribution, then *λ* is a parameter of the exponential distribution and always has a positive value. When the random variables follow an exponential distribution, the expected value and the variance of *X* can be given as 
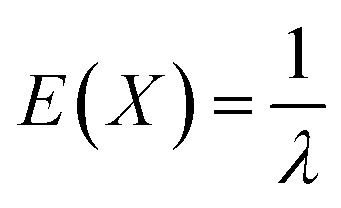
 and 
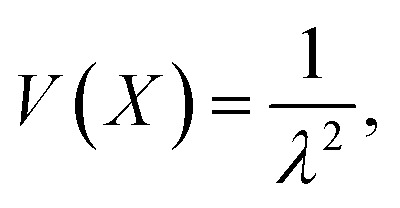
 respectively. The integrated density function derived from this analysis is given by2
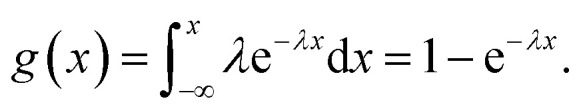


**Fig. 9:**
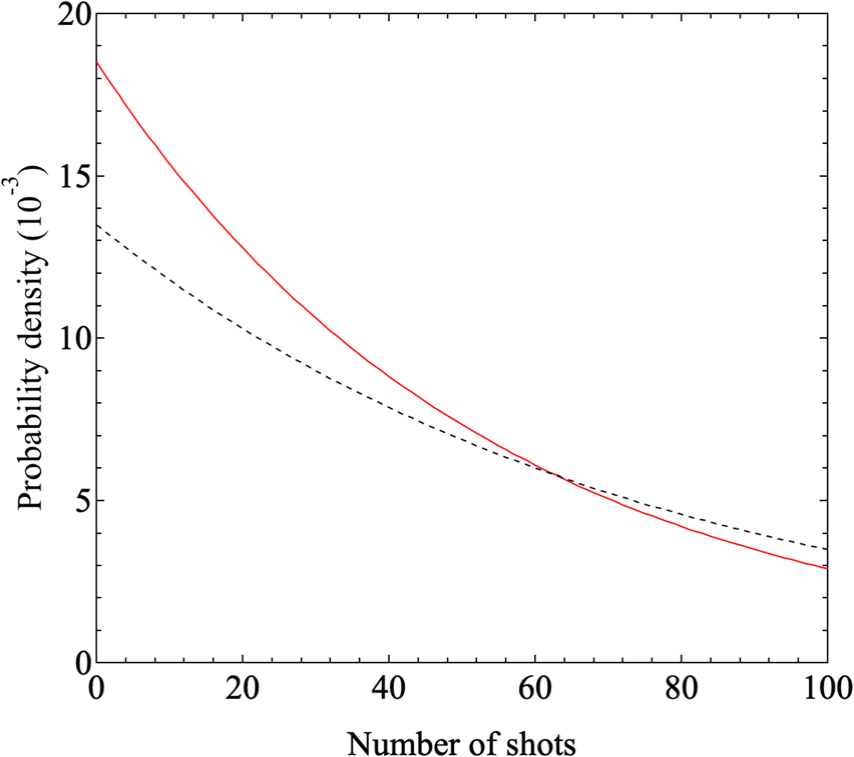
Dependence of the probability density on the number of shots determined by substituting the fitting parameter *λ* estimated from the fitting results shown in Fig. 8 into eqn (1). The red solid and black dashed lines represent the calculated results based on the fitting parameter values of *λ* = 0.0185 and 0.0135 obtained from the printing results with and without homogenization, *i.e.*, with ink ‘B’ and ink ‘A’, respectively.

Because the coverage corresponds to the cumulative probability density, the data can be fitted using the equation 100% × (1 − e^−*λx*^), where *x* represents the number of shots. The fitting results are shown in Fig. 8. The values of *λ* obtained as fitting parameters for the cases with and without the homogenizing treatment were 0.0185 and 0.0135, respectively. The expected values with and without the homogenizer treatment are approximately 54.1 and 74.1 and the corresponding variances are 2.92 × 10^3^ and 5.49 × 10^3^, respectively. Because the value of *λ* is smaller in the case with homogenization, the spatial distribution per discharge, which corresponds to the expected value, is smaller and the variance is also smaller in the homogenization case. Fig. 9 shows the dependence of the probability density on the number of shots, which was obtained by substituting the values given above into the probability density function given by eqn (1). Here, the probability density is the probability that the nanobeacons included per shot are distributed over the paper after printing. A higher number of shots, or trials, corresponds to a lower probability density. This means that even if the number of trials is small, the nanobeacons are still ejected with a specific probability, and as the number of trials increases, then the probability of the nanobeacons being ejected, printed, and distributed decreases. The results presented in Fig. 9 show that the probability density is higher with the homogenized ink when the number of shots is small. Therefore, it is clearly shown that the homogenizing treatment allows the nanobeacons to be dispensed using a smaller number of shots and then diffuse randomly and uniformly over the entire printing area. In other words, these results indicate that the spatial distribution of the nanobeacons is more uniform after inkjet printing and that fewer shots are required to form dots that emit the SERS signal if the homogenization is performed.

**Fig. 10:**
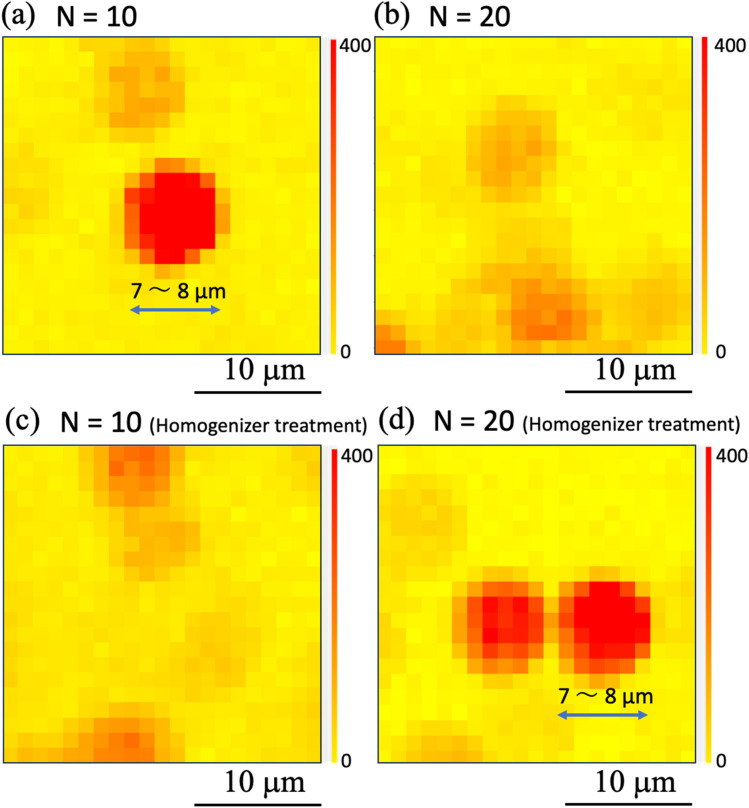
Magnified Raman intensity mapping images obtained by inkjet printing using ink ‘A’ with the number of shots *N* of (a) 10 and (b) 20 and by inkjet printing using ink ‘B’ with the number of shots *N* of (c) 10 and (d) 20. The observation area size of these two mapping images is 25 μm × 25 μm, with measurements being taken at a step size of 1 μm. The Raman mapping images acquired at 1600 cm^−1^ are also shown. The red (yellow) color denotes the high (low) Raman intensity.

**Fig. 11:**
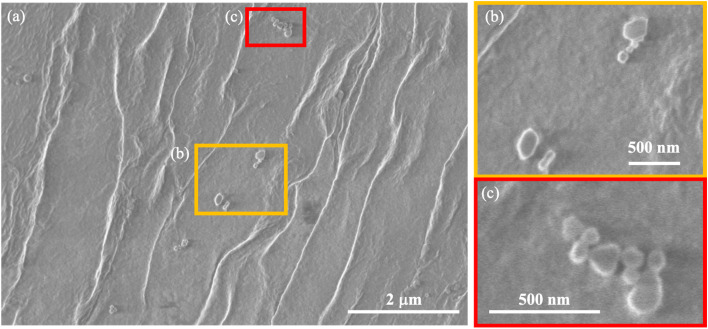
(a) SEM image of the nanobeacons deposited on a paper (b and c) the magnified SEM images of the nanobeacons.

To determine whether the homogenization process changed the SERS signal generation characteristics of the nanobeacons, Raman mapping measurements were taken while using a smaller number of shots and a higher observation magnification. Here, the numbers of shots were fixed at (a), (c) *N* = 10 and (b), (d) *N* = 20 in Fig. 10. The observation results obtained without the homogenizing treatment are shown in Fig. 10(a) and (b), and the printed results obtained with the homogenizing treatment are shown in Fig. 10(c) and (d). Comparison of these results shows that the SERS signal can be observed in the form of a circle with a diameter of approximately 7 to 8 μm, in spite of with and without the homogenizing treatment. Although previous experimental results have shown that the nanobeacon itself has a chain-like structure with a length of approximately 300–500 nm and an irregular shape,^23,24^ when printed in this way and measured *via* the Raman mapping approach, the nanobeacon is observed to form a circle of approximately 7–8 μm in diameter. To elucidate the nanobeacons deposited on a paper, scanning electron microscope (SEM) observation was performed. Fig. 11 shows the SEM image of the nanobeacons on the paper. The nanobeacons were found to be dispersed over the paper as shown in Fig. 11(a). Their shapes shown in the magnified SEM images of Fig. 11(b) and (c) were found to be irregular chain-like structures, consistent with our previous experimental results.^23,24^ The size of the reporter molecules, 4,4′-bypirinde, is about 1.5 nm in the longitudinal direction. The nanobeacon is a structure of multiple gold nanoparticles of several tens nm in size assembled in chains and encapsulating a reporter molecule. Thus, it can be seen in Fig. 11 that the spatial extent of the SERS signal in Fig. 10 does not match the actual size of nanobeacon. The reason for this discrepancy is unclear, but the following possible reasons can be inferred. A reporter molecule is adsorbed at one point in the chain structure of the nanobeacon, which excites SERS because of the cavity effect caused by the chain structure. This cavity-enhanced SERS emission occurs and corresponds to point luminescence. Because the Raman signal from the point luminescence diffuses spherically with a solid angle, it can be inferred that the Raman signal can be observed as a sphere by projecting the spherical luminescence into two dimensions when performing the microscopic Raman mapping, because the detection of the Raman signal is dependent on the spatial resolution of the microscopic Raman spectrometer.^57^

**Fig. 12:**
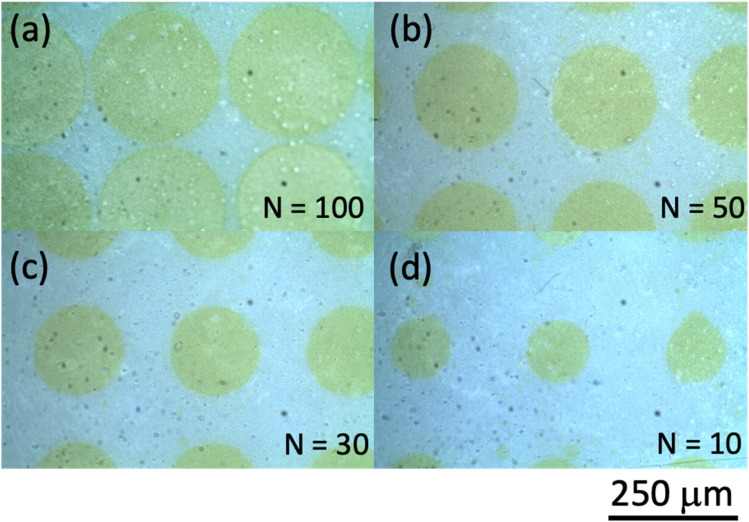
Optical microphotographs of areas printed using ink ‘D’ with the number of shots *N* of (a) 100, (b) 50, (c) 30, and (d) 10. The scale bar length is 250 μm.

**Fig. 13:**
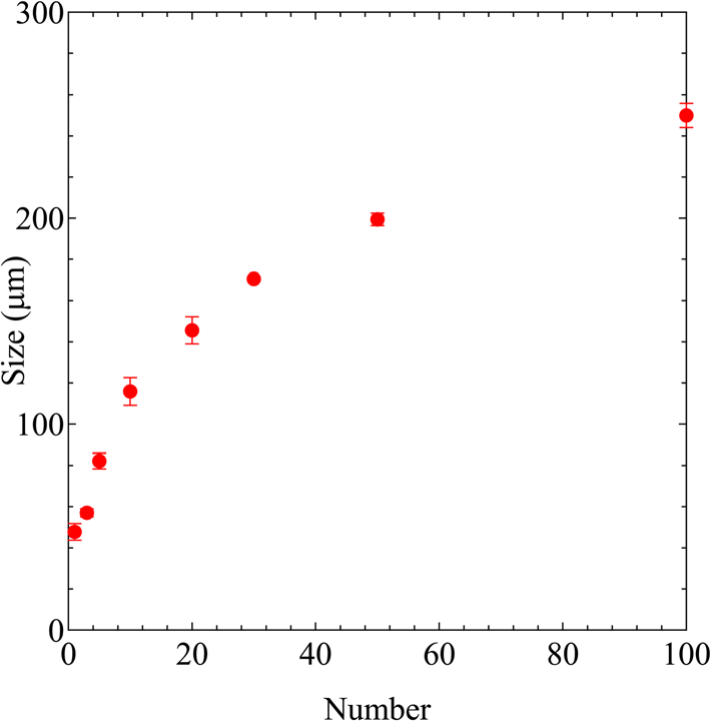
Shot number dependences of dot size (diameter) when printed using ink ‘D’.

Next, printing was performed for inks ‘C’ and ‘D’. In summary, there was little difference between inks ‘C’ and ‘D’ in terms of the characteristics of the inks themselves. Here, we report the results of a study of the dependence of the print diameter of a dot on the number of shots when using ink ‘C’. Fig. 12 shows the dependence of the printing characteristics on the number of shots for ink ‘C’. It is clearly shown that the printed dot diameter increases as the number of shots increases. Fig. 13 summarizes these results and illustrates the dot diameter's dependence on the number of shots. The minimum dot diameter was found to be approximately 50 μm. The reason why this dot diameter increases as the number of shots increases is that, as noted earlier, the size of the entire droplet that is ejected onto the paper increases because the next droplet is stacked on top before the previous droplet has dried.

**Fig. 14:**
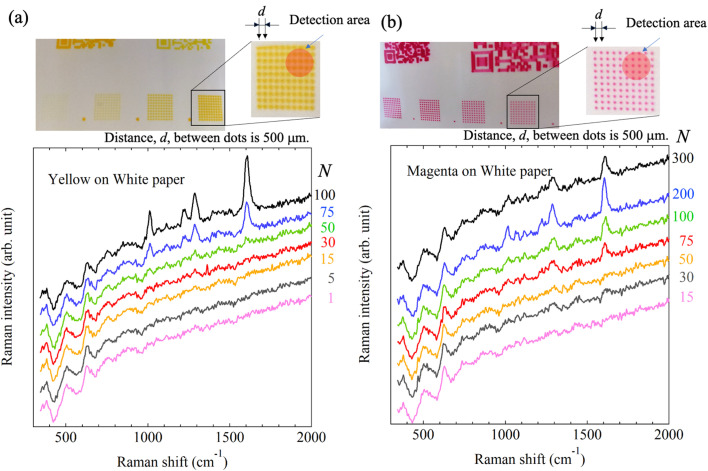
Optical photographs of printing results with inks (a) ‘C’ (yellow) and (b) ‘D’ (magenta) including nanobeacons on paper and the Raman spectra measured using a simple Raman spectrometer. 10 × 10 dots were printed at a pitch of 500 μm. The detection area is displayed schematically in the printed area, as illustrated in the inset of (a). The dot pattern was printed with different numbers of shots *N*. Raman spectra for each number of shots are shown for (a) ink ‘C’ and (b) ink ‘D’.

Next, we printed 10 × 10 dots with a 500 μm pitch while varying the number of shots *N*. Raman detection was performed on these printed dots using a simple mobile Raman spectrometer C13650 (Hamamatsu Photonics K. K., Hamamatsu, Japan, excitation laser wavelength: 785 nm; laser spot diameter: approximately 3 mm). Fig. 14(a) schematically illustrates the relationship between the photograph of the printed material and the object to be measured. The dependence on the number of shots was measured for objects printed using ink ‘C’, and the SERS signal from the nanobeacon could be detected when the number of shots *N* was more than 75. Similarly, Fig. 14(b) shows the results of Raman measurements of objects printed with ink ‘D’. As in the case of ink ‘C’, it was found that the SERS signal was detectable at more than 75 shots. The slanted baseline for the measured spectra was caused by the way in which the printed materials were placed, and it is not related directly to the SERS characteristics. The nanobeacons do not emit the SERS signal of the ink, but only the SERS signal of the molecule that they themselves encapsulate. It is conceivable that the nanobeacons could overlap to create SERS-active nanoscale gaps, which ink-derived molecules could enter and then emit SERS signals, but because the nanobeacons are self-assembled on metastable irregular shapes^23,24^ and do not contact each other sufficiently to create nanogaps between the nanobeacons, SERS generation by the ink is considered to occur very rarely. Because the Raman signal of the ink itself is sufficiently small when compared with that of the nanobeacons, the SERS signal of the nanobeacons is detected over the Raman signal of the ink because of the signal-to-noise ratio (S/N) relationship. Additionally, the Raman signal that originates from the paper, including that from the calcium carbonate and titanium dioxide content, can be detected as the background. However, the SERS signals generated by the nanobeacons are sufficiently stronger than any other signals. As a result, it was shown that even dot-printed materials can be detected adequately using a simple Raman spectrometer if a specific number of nanobeacons are printed.

**Fig. 15:**
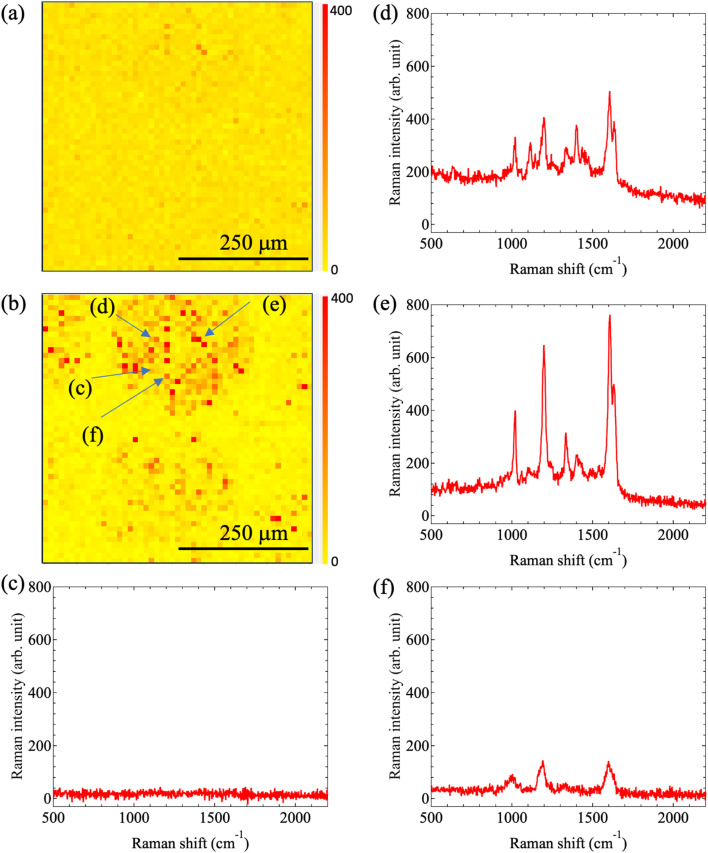
Raman intensity mapping images obtained at Raman shifts of (a) 2000 cm^−1^ and (b) 1600 cm^−1^ in the case of printing using ink ‘C’. The observation area size of these two mapping images is 500 μm × 500 μm, with measurements being taken at a step size of 10 μm. (c)–(f) SERS spectra for the locations in the Raman mapping image shown in (b).

Finaly, Fig. 15 shows the Raman mapping image results obtained after inkjet printing with ink ‘C’, which included nanobeacons dispersed in the yellow ink. The Raman mapping was performed for dots printed using 100 shots. The Raman mapping images acquired at 2000 cm^−1^ and 1600 cm^−1^ are shown in Fig. 15(a) and (b), respectively. In the Raman mapping image acquired at 2000 cm^−1^ in Fig. 15(a), the printed dot pattern was not observable. In contrast, in the Raman mapping image acquired at 1600 cm^−1^ in Fig. 15(b), the printed dot pattern is clearly observable. We found that there were mixtures of strong and weak Raman signal areas within the dots. This is almost the same result that was observed for water-based inks ‘A’ and ‘B’ described above. The SERS spectra acquired at each location are shown in Fig. 15(c)–(f). Initially, we focus on the background Raman signal from the printed area in Fig. 15(b). At location (c) in Fig. 15(b), a flat spectrum was detected as shown in Fig. 15(c), indicating that there were no nanobeacons present and that the background signal derived from the yellow ink itself was not detectable. At location (d), a relatively small Raman spectrum was detected, and this spectrum is shown in Fig. 15(d).

At locations (d)–(f), the spectra that originated from the nanobeacons were detectable, as shown in Fig. 15(d)–(f), respectively. Although there are differences among the magnitudes of the Raman intensity for each spectrum, the peak positions that originate from the basic molecular vibrations remain almost unchanged. This indicates that nanobeacons are present at these locations. One possible cause of the differences in Raman intensity is that the nanobeacons are printed on paper, which means that the focal distance to the microscope may be changed by stacking of the beacons on top of each other or encapsulation of the beacons in the paper fibers. Another possibility is that the laser excitation and the Raman emission properties may be modulated by the variations in the properties of the nanobeacons, the paper fibers, and other materials. In any case, we were able to demonstrate that the nanobeacons are distributed within the printed dots and that they can emit SERS signals. It was also found that the nanobeacons can be printed as an ink, even when they have been mixed with a water-based ink, without losing the properties of the nanobeacons.

In view of the results above, it was found that the nanobeacon solution can be mixed with water-based inks that have been colored using dyes without deactivating the nanobeacon function. This means that there can be more variations in the types of inks that can contain nanobeacons, and it can also be said that even if it appears that the ink is printed with the same color, it is actually the ink in which the nanotag is hidden. This study will be expected to be subject to further development to provide authentication systems based on nanotags printed using inkjet printers and thus realize Society 5.0 (ref. 69) and digital product passports.^70^

## Conclusion

In this study, inkjet printing was performed using inks that contained nanobeacons and the printing properties of these inks were investigated in terms of their shot number dependences and their micro-Raman mapping properties. We found that: (1) use of an ultrasonic homogenizer resulted in more uniform spatial distributions for the nanobeacons when printed, and it also enabled printing using fewer shots; (2) the signals from the nanotags were observed as circles with diameters of 7 to 8 μm *via* the micro-Raman system used in this study; and (3) the nanobeacons were observed as a single nanotag with a diameter of approximately 7 to 8 μm *via* the micro-Raman system used in this study. The signals from the nanotags were also observed in the form of circles with diameters of approximately 7 to 8 μm. This behavior may be caused by the fact that the reporter molecules encapsulated within the nano-assembly function as a point light source. (4) It was also confirmed that it is possible to mix the nanobeacons into a water-based ink and then print them *via* inkjet printing.

The application of inkjet printing and the inkjet printing of nanobeacons reported in this research is expected to lead to the evolution of nanotags that can carry even more information in addition to the determination of authenticity provided by the nanobeacons.

## Data availability

All data supporting this article have been included in this manuscript.

## Conflicts of interest

There are no conflicts to declare.
